# Jejunal Enterotomy for Specimen Retrieval in D-LECS: A Case Report

**DOI:** 10.70352/scrj.cr.25-0544

**Published:** 2025-12-20

**Authors:** Kota Okuno, Hiroki Harada, Takuya Wada, Gen Kitahara, Shohei Fujita, Tadashi Higuchi, Mikiko Sakuraya, Koshi Kumagai, Yusuke Kumamoto, Takeshi Naitoh, Keishi Yamashita, Chika Kusano, Naoki Hiki

**Affiliations:** 1 Department of Upper Gastrointestinal Surgery, Kitasato University School of Medicine, Sagamihara, Kanagawa, Japan; 2 Department of Gastroenterology, Kitasato University School of Medicine, Sagamihara, Kanagawa, Japan; 3 Department of General-Pediatric-Hepatobiliary Pancreatic Surgery, Kitasato University School of Medicine, Sagamihara, Kanagawa, Japan; 4 Department of Lower Gastrointestinal Surgery, Kitasato University School of Medicine, Sagamihara, Kanagawa, Japan; 5 Division of Advanced Surgical Oncology, Research and Development Center for New Medical Frontiers, Kitasato University School of Medicine, Sagamihara, Kanagawa, Japan

**Keywords:** duodenal adenoma, D-LECS, specimen retrieval, enterotomy, minimally invasive surgery

## Abstract

**INTRODUCTION:**

Laparoscopic and endoscopic cooperative surgery for duodenal tumors (D-LECS) combines endoscopic submucosal dissection (ESD) with laparoscopic reinforcement, offering a minimally invasive option for superficial non-ampullary duodenal tumors. However, oral retrieval of resected specimens may be difficult for large or firm tumors, risking fragmentation and compromised pathological assessment.

**CASE PRESENTATION:**

We report a case involving a 42-year-old woman with a 40-mm villous tumor in the second portion of the duodenum. Due to the size of the tumor, D-LECS (ESD with laparoscopic reinforcement) was contemplated. After successful ESD, the specimen could not be retrieved orally due to non-passage through the pyloric ring. To preserve *en bloc* integrity, the specimen was advanced into the jejunum, and the jejunal loop was exteriorized through a small umbilical incision. An enterotomy was performed extracorporeally, allowing safe retrieval without contamination. The enterotomy was securely closed, and the patient recovered without complications.

**CONCLUSIONS:**

This case demonstrates that jejunal exteriorization and controlled enterotomy is a safe and practical alternative in D-LECS when oral extraction is unfeasible, enabling accurate pathological evaluation while preserving minimally invasive benefits.

## INTRODUCTION

Duodenal tumors are rare and often detected incidentally during routine upper gastrointestinal endoscopy.^1)^ Among these, superficial non-ampullary duodenal epithelial tumors (SNADETs) are generally considered suitable for local resection due to their low malignant potential.^2,3)^ Endoscopic mucosal resection (EMR) is effective for small lesions; however, its R0 resection rate decreases with increasing tumor size, often necessitating alternative approaches such as segmental resection. Conversely, surgical procedures like pancreaticoduodenectomy offer curative potential but are highly invasive and associated with significant morbidity.^4,5)^ Endoscopic submucosal dissection (ESD) enables *en bloc* resection of large lesions, but the thin duodenal wall and narrow lumen pose a considerable risk of delayed perforation.^6,7)^ To mitigate these risks, laparoscopic and endoscopic cooperative surgery for duodenal tumors (D-LECS) has been developed. This technique combines ESD with laparoscopic reinforcement and has shown promise in improving both safety and oncological outcomes.^8,9)^ However, in cases involving large or firm lesions, oral retrieval of the resected specimen may be technically unfeasible. Here, we report a case in which safe *en bloc* retrieval was achieved by advancing the specimen into the jejunum, exteriorizing the jejunal loop through a small umbilical incision, and performing a controlled enterotomy outside the abdominal cavity. This case highlights a practical and safe alternative for specimen retrieval in D-LECS.

## CASE PRESENTATION

**Fig. 1:**
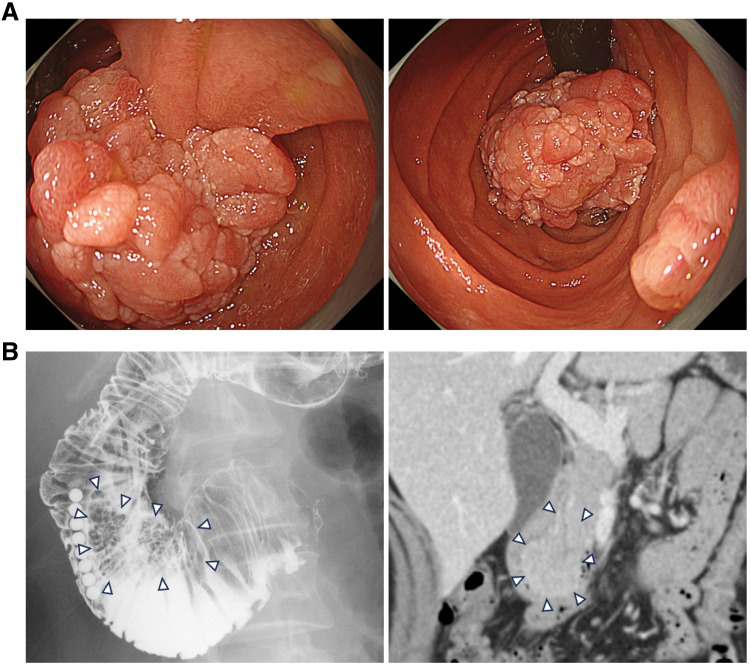
Preoperative endoscopic and radiological findings. (**A**) Esophagogastroduodenoscopy showing a 40-mm villonodular tumor in the 2nd portion of the duodenum (left: close-up view; right: tumor occupying the lumen). (**B**) Upper gastrointestinal contrast study demonstrating a filling defect (left, arrowheads) and CT revealing a corresponding lesion without local invasion or distant metastasis (right, arrowheads).

A 42-year-old woman with a history of cervical cancer and no prior abdominal surgeries underwent an upper gastrointestinal endoscopy for screening purposes, during which a duodenal tumor was detected. She was referred for further treatment. The patient was not currently taking any medications and had no known allergies. During esophagogastroduodenoscopy (EGD), a 40-mm villonodular lesion was identified in the second part of the duodenum and classified as type 0-Ip (**Fig. 1A**, left panel). The lesion occupied the lumen (**Fig. 1A**, right panel). Biopsy was avoided to prevent fibrosis. Upper gastrointestinal contrast study revealed a filling defect at the lesion site (**Fig. 1B**, left panel). CT confirmed a 40-mm tumor at the same site, with no evidence of local invasion or distant metastasis (**Fig. 1B**, right panel).

**Fig. 2:**
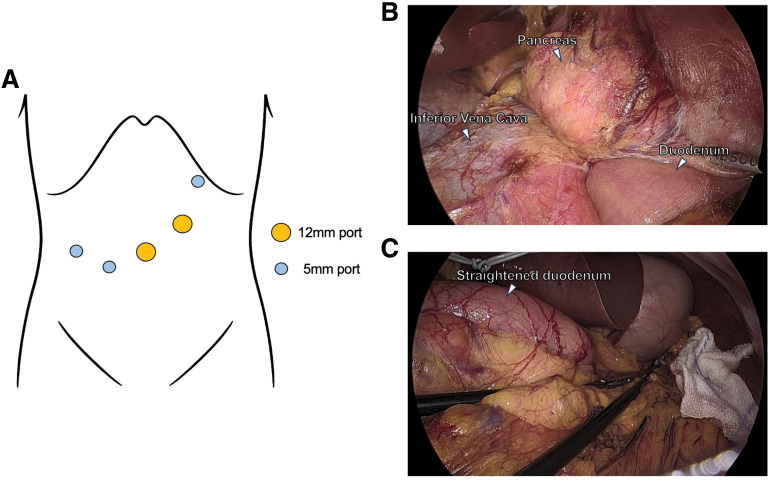
Port placement and laparoscopic findings. (**A**) Schema of port placement: two 12-mm ports (yellow) and three 5-mm ports (blue). (**B**) Operative view after Kocher maneuver, showing exposure of the pancreas, inferior vena cava, and duodenum. (**C**) Straightened duodenum achieved after mobilization.

D-LECS was planned. As shown in **Fig. 2A**, 5 laparoscopic ports were placed. The omental bursa (bursa omentalis) was opened, the transverse colon and ascending colon were mobilized, and the Kocher maneuver was performed (**Fig. 2B**). Adequate visualization was achieved, and the duodenum was straightened (**Fig. 2C**). Endoscopic resection was initiated, and an atraumatic intestinal clamp was temporarily placed approximately 5 cm distal to the ligament of Treitz to retain fluid in the lumen. ESD using water pressure method^10)^ was performed, and the tumor was resected *en bloc* without intraoperative complications. However, due to the size and hardness of the specimen, extraction through the pyloric orifice failed.

**Fig. 3:**
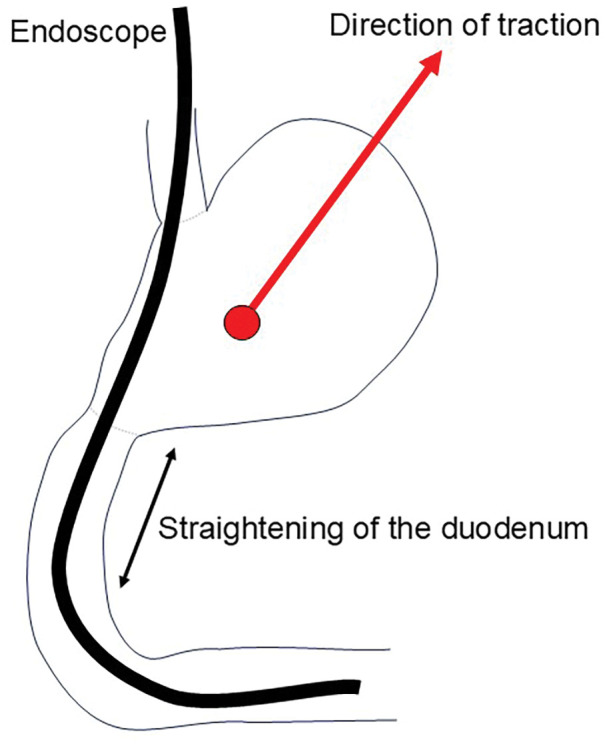
Straightening of the duodenum for endoscopic advancement. Schematic illustration showing straightening of the duodenum to facilitate smooth passage of the endoscope through the 2nd portion of the duodenum. The red arrow indicates the direction of gastric traction.

The clamp was left in place approximately 30 cm distally from the Treitz ligament. Under endoscopy, the specimen was grasped with a retrieval net and advanced into the jejunum proximal to the clamp. Straightening the duodenum as much as possible allowed the endoscope to reach the jejunum. A schematic illustration (**Fig. 3**) showing duodenal straightening is provided, and an intraoperative endoscopic video demonstrating the specimen transfer is available (**Supplementary Video 1**). To fix the tumor within the jejunal segment, another clamp was placed proximal to the resected specimen. The jejunal loop containing the specimen was extracted through a small umbilical incision. A semicircular incision was made along the short axis of the jejunal wall, and the specimen was completely removed. The intestinal incision was closed with a full-thickness continuous suture using 4-0 PDS. A video of specimen retrieval is available (**Supplementary Video 2**). The interval from specimen resection to the start of transfer was 19 minutes, and transfer was completed at 22 minutes. After confirming the absence of residual tumor, specimen retrieval was completed at 47 minutes, and suturing was finished at 55 minutes. The ESD site of the duodenum was reinforced with 4 horizontal mattress sutures using 3-0 Monocryl to prevent postoperative leakage and stricture. A drain was inserted into the foramen of Winslow. The total operative time was 253 minutes, with minimal bleeding.

The patient resumed oral intake on POD 2. She had no postoperative fever or signs of leakage. The patient was discharged without complications on POD 8. Final pathological examination revealed a 35-mm, low-grade tubular adenoma with no evidence of infiltration. The lateral and vertical resection margins were negative.

## DISCUSSION

To clarify the clinical context of this case, a literature search was conducted using PubMed. The search period was from January 2008 to August 2025, with the keywords “duodenal LECS” and “D-LECS”. Only English original and review articles were included, and studies describing indications, lesion size criteria, retrieval routes, and complications were prioritized.

Laparoscopic and endoscopic cooperative surgery (LECS), including D-LECS, is a minimally invasive technique for that enables safe resection of superficial non-ampullary duodenal tumors by combining ESD with laparoscopic reinforcement.^8,11)^ D-LECS has 2 main approaches: laparoscopic-assisted full-thickness resection (FTR with D-LECS) and laparoscopic-assisted ESD (ESD with D-LECS). Among these, ESD with D-LECS is preferred because it minimizes the risk of duodenal fluid leakage and tumor exposure to the peritoneal cavity.^9)^

During D-LECS, it is generally recommended to avoid opening the intestinal tract into the abdominal cavity during specimen retrieval to reduce the risk of intra-abdominal infection or tumor cell dissemination. Consequently, specimens are typically retrieved orally via narrow channels such as the pyloric ring or the esophagogastric junction. Large or firm tumors may not pass through these routes. Although fractionating and retrieving specimens is technically feasible, it carries the risk of incomplete retrieval within the intestinal tract and may compromise pathological assessment. In the stomach, specimens can be retrieved through a small abdominal incision after full-thickness gastric wall incision, but there have been no reports of unrecoverable specimens originating from the duodenum. According to the European Society of Gastrointestinal Endoscopy (ESGE) clinical guideline,^12)^ while there are no data on passage rates by size, specimens exceeding 2.0–2.5 cm may not pass through the pyloric ring. Therefore, depending on the tumor's hardness, alternative retrieval methods should be considered for specimens with a short diameter exceeding 2 cm, as they may not pass through the pyloric ring.

In the present case, an initial attempt was made to retrieve the resected specimen orally. At our institution, the indication for oral specimen retrieval is principally an upper diameter limit of 30 mm. Tumor consistency is also considered in the decision-making process, and softer lesions are more likely to pass the pylorus and are therefore more suitable for oral extraction. Although the lesion in this case exceeded 30 mm, it was suspected to be an adenoma and felt soft on assessment, so oral retrieval was attempted first. For procedures that presuppose oral retrieval, such as non-exposed endoscopic wall-inversion surgery (NEWS) or Closed-LECS, we similarly adopt 30 mm as the practical upper limit for candidate lesions. When passage through the pyloric ring proved difficult, the resected specimen was advanced endoscopically into the jejunum to maintain *en bloc* integrity. Approximately 30 cm distal to the ligament of Treitz, the jejunal loop was exteriorized via a laparoscopic port, and a controlled enterotomy was performed outside the abdominal cavity to retrieve the specimen intact. As illustrated in the schematic (**Fig. 3**), sufficient Kocher’s mobilization and straightening of the duodenum enabled the endoscope to reach the 2nd portion of the duodenum without looping, thereby allowing smooth advancement into the jejunum. Although a previous report^13)^ described that Kocher’s mobilization and duodenal extension facilitate endoscopic manipulation in the duodenum, there are no comparative data directly verifying that these maneuvers facilitate access to the jejunum. Therefore, this interpretation is considered a literature-based rational inference.

This technique is considered feasible in cases where the duodenum can be sufficiently straightened and Kocher’s mobilization can be safely performed. This technique avoided contamination of the abdominal cavity, enabled secure closure of the enterotomy, and preserved the specimen’s integrity for accurate histopathological evaluation. Closure of the externalized jejunal incision was straightforward and complication-free. By contrast, direct duodenal wall resection carries a risk of postoperative complications, including stricture and intra-abdominal infection, especially when full-thickness closure is necessary.^9)^

To our knowledge, this is the first reported case of intentional specimen retrieval via jejunal enterotomy in D-LECS. The technique demonstrates a novel and feasible solution for managing cases in which oral retrieval is not possible.

## CONCLUSIONS

When oral retrieval of resected specimens is not feasible in D-LECS, externalized jejunal enterotomy offers a safe and practical alternative. This approach preserves *en bloc* integrity, avoids intra-abdominal contamination, and allows accurate pathological assessment. It may be a useful option for selected cases involving large duodenal tumors.

## SUPPLEMENTARY MATERIALS

Supplementary Video 1 Under endoscopic guidance, the specimen was grasped with a retrieval net and advanced through the straightened duodenum into the jejunum proximal to the intestinal clamp.

Supplementary Video 2 Specimen retrieval via extracorporeal jejunal enterotomy. The resected specimen was advanced endoscopically into the jejunum, exteriorized through a small umbilical incision, and retrieved via a controlled enterotomy outside the abdominal cavity.

## DECLARATIONS

### Availability of data and materials

All the data in this study are included in this article.
